# An Ancient Chinese Herbal Decoction Containing Angelicae Sinensis Radix, Astragali Radix, Jujuba Fructus, and Zingiberis Rhizoma Recens Stimulates the Browning Conversion of White Adipocyte in Cultured 3T3-L1 Cells

**DOI:** 10.1155/2019/3648685

**Published:** 2019-06-16

**Authors:** Guowei Gong, Guangyi Han, Huan He, Tina T. X. Dong, Karl W. K. Tsim, Yuzhong Zheng

**Affiliations:** ^1^Department of Bioengineering, Zunyi Medical University, Zhuhai Campus, Zhuhai, Guangdong, 519041, China; ^2^Gansu Institute for Drug Control, Lanzhou, Gansu, 730070, China; ^3^Shenzhen Key Laboratory of Edible and Medicinal Bioresources, SRI, The Hong Kong University of Science and Technology, Shenzhen, 518057, China; ^4^Division of Life Science, Center for Chinese Medicine, The Hong Kong University of Science and Technology, Clear Water Bay, Hong Kong; ^5^Department of Biology, Hanshan Normal University, Chaozhou, Guangdong 521041, China

## Abstract

**Background:**

Abnormal storage of white adipocyte tissue (WAT) is the major factor causing obesity. The promising strategies for obesity treatment are building up the brown adipocyte tissue (BAT) and/or expedite fatty acid catabolism. Traditional Chinese Medicine (TCM) sheds light on preventing obesity. Ginger is one of the most effective herbs for antiobesity by accelerating browning WAT. To fortify the antiobesity effect of ginger, an ancient Chinese herbal decoction composed of four herbs, Angelicae Sinensis Radix (ASR), Astragali Radix (AR), Jujuba Fructus (JF), and Zingiberis Rhizoma Recens (ZRR; ginger), was tested here: this herbal formula was written in AD 1155, named as Danggui Buxue Tang (DBT_1155_). Therefore, the antiobesity function of this ancient herbal decoction was revealed* in vitro* by cultured 3T3-L1 cells.

**Materials and Method:**

The lipid accumulation was detected by Oil Red O staining. Furthermore, the underlying working mechanisms of antiobesity functions of DBT_1155_ were confirmed in 3T3-L1 cells by confocal microscopy, western blot, and RT-PCR.

**Results:**

DBT_1155_ was able to actuate brown fat-specific gene activations, which included (i) expression of PPAR*γ*, UCP1, and PCG1*α* and (ii) fatty acid oxidation genes, i.e., CPT1A and HSL. The increase of browning WAT, triggered by DBT_1155_, was possibly mediated by a Ca^2+^-AMPK signaling pathway, because the application of Ca^2+^ chelator, BAMPTA-AM, reversed the effect.

**Conclusion:**

These findings suggested that the herbal mixture DBT_1155_ could potentiate the antiobesity functions of ginger, which might have potential therapeutic implications.

## 1. Introduction

Obesity is characterized as abnormal or excessive accumulated adipose tissues, which is believed to be induced by multiple factors, including genetically and environmentally. Obesity incidence is increasing and becomes a normal phenomenon in both developing and developed countries, posting a great challenging for health care professionals. The obese persons could undergo high risks of metabolic abnormalities, diabetes, and several types of cancers diseases [1, 2]. Antiobesity therapeutic treatments have been proposed for decades. The limitation of carbohydrate intake used to be believed as the most effective strategy for antiobesity; however, this treatment has been reported to have negative impact on mental development [3, 4]. On the other hand, the side effects of popular weight loss synthetic medicines, e.g., phentermine-topiramate and lorcaserin, are commonly ameliorating the risks of hepatorenal syndrome and resulting in reducing the patient's life quality [5].

There are two types of adipose tissues found within human body, i.e., white adipose tissues (WAT) and brown adipose tissues (BAT). The major functions of WAT are heating insulation, buffering mechanical cushion, and, finally, storing of energy. WAT is acting as fuel for energy imbalances when the intaking energy is smaller than outputting energy; therefore, WAT is considered as a crucial component in contributing obesity [6]. BAT, on the other hand, accelerates energy expenditure and finally combats obesity [7, 8]. Physical exercise is one of typical routines to lose weight and reshape the body by hastening WAT browning and stimulating fatty acid oxidation [9]. The high expression level of mitochondrial uncoupling protein 1 (UCP1) is a hallmark of browning WAT [9]. Furthermore, peroxisome proliferator-activated receptor (PPAR*γ*) and peroxisome proliferator-activated receptor-gamma coactivator 1 (PGC1*α*) are two transcriptional factors in modulating adipogenous-related gene expressions, which are highly expressed in BAT [10]. On the other hand, carnitine palmitoyl transferase I A (CPT1A) and hormone-sensitive lipase (HSL) genes can enhance mitochondrial activities and stimulate fatty acid oxidation, and therefore they are classified as the signature of fatty acid oxidation [11].

There is an increasing consumption of functional foods or food supplements aiming to control weight. Traditional Chinese Medicine (TCM) has drawn attention in the market; because it has a peculiar and organized understanding of obesity according to its specific concept. The major determinant eliciting obesity is the imbalance of “Yin” and “Yang,” which results in stagnation of “Qi” and “Blood” [12]. Most of synthetic medicines alter the interaction of neurological and/or hormonal signals in acting as appetite suppressants or as inducers of diarrhea, which could be damaging to psychology and physiology of our bodies [13]. Zingiberis Rhizoma Recens (ZRR, root of* Zingiber officinale* Roscoe; ginger) is one of the most popular spices utilized in the world, and its antiobesity function has been widely reported [14]. The intake of ginger extract or curcumin, one of bioactive constituents found within ginger, significantly reduced body weight, leptin, insulin, amylase, lipase plasma, and tissue lipids in rats. In parallel, the level of peroxisomal catalase in serum was enhanced in ginger- and curcumin-treated rats [15–18]. ZRR was able to activate AMPK pathway, the key signaling in modulating WAT browning [19]. On the other way, oral administration of ZRR in human could reduce hunger sensation [15]. In TCM formulation, ginger is being included commonly in many herbal formulae, and indeed the therapeutic functions of these herbal formulae are believed to enhance thermogenesis, as such to reduce obesity.

An ancient herbal mixture, written by* Chen Suan* of Song Dynasty (1155 AD) in* “Chensuan Fuke Buji”*, is known to improve “Qi” and “Blood,” named as Danggui Buxue Tang (DBT_1155_). DBT_1155_ composes four herbs: Angelicae Sinensis Radix (ASR), Astragali Radix (AR), Jujuba Fructus (JF), and Zingiberis Rhizoma Recens (ZRR) in a weight ratio of 36: 30: 15: 20. The antiobesity functions of curcumin-enriched ZRR were widely reported, and this herbal formula was shown to have antilipid accumulation in our preliminary study. Thus, the antiobesity functions of DBT_1155_ in cultured 3T3-L1 adipocytes were tested here.

## 2. Materials and Methods

### 2.1. Preparation of Herbal Extract

The raw herbs of root of* Astragali membranaceus* var.* mongholicus* (AR), root of* Angelica sinensis *(Oliv) Diels. (ASR), fruit of* Ziziphus jujuba* cv. Jinsixiaozao (JF), and rhizome of* Zingiber officinale* Roscoe (ZRR; ginger) were collected and identified in 2013. The voucher specimen of AR, ASR, JF, and ZRR was kept in Centre for Chinese Medicine of HKUST. AR, ASR, JF, and ZRR in a weight ratio of 36: 30: 15: 20 were used to prepare DBT_1155_ decoction. The mixture was boiled in 8 volumes of water for twice. Fifty grams of ZRR was also boiled in water twice, each with 8 volumes of water. This preparation was verified in previous studies [20, 21]. All samples were dried by lyophilization and resuspended in water at final concentration of 100 mg/mL, which were kept at −80°C.

### 2.2. HPLC Analysis and Chemical Quantifications

Chemical standardization and quantification of herbal mixture are the first step in performing biological assay [20, 22, 23]. According to China Pharmacopeia, ferulic acid was chosen as marker chemical in ASR. Calycosin and formononetin were selected as quantification markers in AR; cyclic AMP (cAMP) was reported to be the bioactive chemical found within JF; and 6-gingerol was elite as standard for ZRR (CP, 2015). The HPLC mobile phases were composed of 0.1% formic acid in water (A) and 0.1% formic acid in acetonitrile (B), respectively. An elution gradient was set up as follows: 0-2 min isocratic gradient 95% (A); 2-4 min, linear gradient 95-90% (A); 4-15 min, linear gradient 90-80% (A); 15-20 min, isocratic gradient 80% (A); 20-27 min, linear gradient 80%-70% (A); and 17-70 min, linear gradient 70-45% (A). The preequilibration period of 15 min was used between each run. The column temperature was set to 25°C. The injection volume was 10 *μ*L. A wavelength of 254 nm was employed for detection. The flow rate was set at 1.0 mL/min. Agilent RRLC 1200 series system (Waldron, Germany) equipped with a degasser, a binary pump, an auto-sampler, a diode array detector (DAD), and a thermo-stated column compartment was adopted for establishment of fingerprint for herbal extracts. The HPLC condition was conducted on Agilent ZORBAX SB-Aq (4.6 × 250 mm, 5 *μ*m) C18 column.

### 2.3. Cell Cultures

Mouse 3T3-L1 fibroblast cells (CL-173) were obtained from ATCC (Manassas, VA) and maintained at 37°C in a water-saturated incubator containing 5% CO_2_ and in DMEM supplemented with 4.5 g/L glucose, 10% FBS, 100 U/mL penicillin, and 100 *μ*g/mL streptomycin. Induction of lipogenic differentiation was detailed in a previous study [24]. Briefly, cultured cells were treated with dexamethasone (1 *μ*M, Sigma-Aldrich, St Louis, MO), insulin (1.8 *μ*M, Sigma-Aldrich), and dibutryl-cAMP (300 *μ*M, Sigma-Aldrich) for 72 hours to induce lipogenesis. The cultures were set as day 0 and replaced with the culture medium containing insulin (1.8 *μ*M) for every two days. At day 10, about 80% of cultures were induced to contain triglyceride. Treatments including negative control (0.02% DMSO only), cocktail (1.8 *μ*M of rosiglitazone and triiodothyronine), low concentration of DBT (DBT-L, 0.125 mg/mL), and high concentration of DBT (DBT-H, 1.0 mg/mL) were given to differentiated cultures (on day 10) for 72 hours. Unless described otherwise, all the culture reagents were purchased from Invitrogen Technologies (Waltham, MA).

### 2.4. Cell Viability

The cell viability was measured by MTT assay. In brief, cells were cultured in 96-well plate. After drug treatments for indicated durations, MTT solution was added into the cultures in the final concentration of 0.5 mg/mL; after incubation for 2 hours, the production of purple crystal was dissolved by DMSO solvent. The absorbance at 570 nm was measured.

### 2.5. Oil Red O Staining

Oil Red O at 0.2% in isopropanol was filtered. Experimental cultured cells were washed with PBS, fixed by paraformaldehyde (4% in PBS, Sigma-Aldrich) for 5 min, incubated with Oil Red O staining for 30 min, and washed twice with PBS. The stained triglyceride (TG) was resolved in isopropanol and measured at the absorbance of 490 nm [24].

### 2.6. Laser Confocal Fluorescence Microscopy

Fluorimetric measurements were performed on cultured 3T3 cells using an Olympus Fluoview FV1000 laser scanning confocal system (Olympus America, Manassas, VA) mounted on an inverted Olympus microscope, equipped with a 10X objective. Intracellular Ca^2+^ concentration was detected by fluorescent calcium indicator Fluo-4 AM (Sigma-Aldrich). Cultured cells were seeded on the glass coverslips and incubated for 30 min at 37* *°C in a normal physiological solution containing Ca^2+^-free normal physiological solution containing 5 *μ*M Fluo-4 AM. A23187 (Sigma-Aldrich), a calcium ionophore, was used as a positive control. The amount of Ca^2+^ was evaluated by measuring the fluorescence intensity exiting at 488 nm and emitted at 525 nm.

### 2.7. Western Blot Assay

The protein expressions of PPAR*γ*, PGC1*α*, UCP1, and internal control GAPDH were revealed by western blot. Cultures were seeded onto 6-well plate. After drug treatment for 72 hours, including inhibitor application, the cultures were harvested in high salt lysis buffer (1 M NaCl, 10 mM HEPES, pH 7.5, 1 mM EDTA, 0.5% Triton X-100), followed by centrifugation at 16,100 rpm for 10 min at 4°C. Samples with equal amount of total protein were added with 2X lysis buffer (0.125 M HCl, pH 6.8, 4% SDS, 20% glycerol, 2% 2-mercaptoethanol and 0.02% bromophenol blue) and heated to 95°C, and the protein was subjected to SDS-PAGE analysis. After transferring, the membranes were incubated with antibodies against PPAR*γ*, PGC1*α*, UCP1, and GAPDH (CST, Danvers, MA) at 1: 3,000 dilutions at cold room overnight.

The phosphorylation of AMPK was also determined by western blot assay. Differentiated cultures were serum-starved for 3 hours before the drug application. After treatment with BAMPTA-AM (10 *μ*M) or WZ4003 (100 nM; Selleck, Munich, Germany), the cultures were collected immediately in lysis buffer (125 mM Tris–HCl, 2% SDS, 10% glycerol, 200 mM 2-mercaptoethanol, pH 6.8). The protein was subjected to SDS-PAGE analysis. After transferring the proteins to membranes, the membranes were incubated with anti-phospho-AMPK (Cell Signaling, MA) at 1: 5,000 dilution and anti-total-AMPK (Cell Signaling) at 1: 5,000 dilution at 4°C for 12 hours. Following incubation in horseradish peroxidase- (HRP-) conjugated anti-rabbit secondary antibodies in 1: 5,000 dilution for 3 hours at room temperature, the immune-complexes were visualized by the enhanced chemiluminescence (ECL) method (Amersham Biosciences, Piscataway, NJ). The band intensities in the control and agonist-stimulated samples, run on the same gel and under strictly standardized ECL conditions, were compared on an image analyzer, using in each case a calibration plot constructed from a parallel gel with serial dilutions of one of the samples.

### 2.8. RT-PCR Analysis

Total RNA was extracted from 3T3-L1 adipocyte cells with RNAzol reagent (Invitrogen) according to manufacturer's instructions. RNA samples with OD260/OD280 ratio higher than 2.0 were employed for PCR. One *μ*g of total RNA was employed for the production of cDNA, using a PCR system. The oligonucleotide primer sequence was as follows: peroxisome proliferator-activated receptor (PPAR*γ*): 5′-CCA GAG TCT GCT GAT CTG CG-3′ and 5′-GCC ACC TCT TTG CTC TGA TC-3′; peroxisome proliferator-activated receptor *γ* coactivator 1 (PGC1*α*): 5′-GAC CTG GAA ACT CGT CTC CA-3 and 5′-AAA CTT GCT AGC GGT CCT CA-3′; carnitine palmitoyl transferase I A (CPT1A): 5′-GGA CAT TAT CAC CTT GTT TGG C-3′ and 5′-GGA GCA ACA CCT ATT CAT T-3′; hormone-sensitive lipase (HSL): 5′-GCG CTG GAG GAG TGT TTT T-3′ and 5′-CGC TCT CCA GTT GAA CCA AG-3′; mitochondrial uncoupling protein 1 (UCP1): 5′-GAT GGT GAA CCC GAC AAC TT-3′ and 5′-CTG AAA CTC CGG CTG AGA AG- 3′; 18S: 5′-GTA ACC CGT TGA ACC CCA TT-3′ and 5′-CCA TCC AAT CGG TAG TAG CG-3′. Transcript levels were quantified by using _Δ_Ct value method, where the values of target genes were normalized by 18S in the same sample at first before comparison. PCR products were analyzed by gel electrophoresis and melting curve analysis, as to confirm the specific amplification.

### 2.9. Statistical Analysis and Other Assays

Protein concentrations were measured by Bradford's method (Herculues, CA). Statistical tests have been done by using one-way analysis of variance. Data were analyzed by t-test and expressed as Mean ± SEM. Statistically significant changes were classified as significant (*∗*) where* p* < 0.05, more significant (*∗∗*) where* p *< 0.01, and highly significant (*∗∗∗*) where* p* < 0.001 as compared with control group.

## 3. Results

### 3.1. Chemical Standardization of DBT_*1155*_

**Figure 1:**
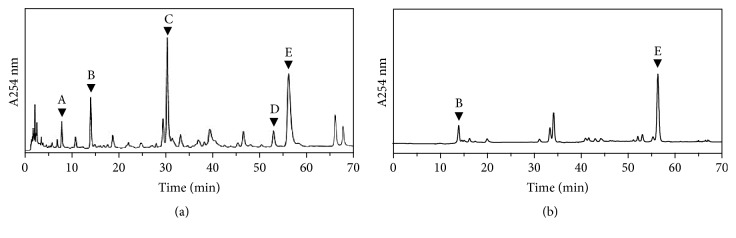
*Typical chromatograms of DBT*_*1155*_ *and ZRR extracts*. Ten *μ*L of 100 mg/mL of DBT_1155_ decoction (a) and 100 mg/mL of ZRR extract (b) were subjected to HPLC-DAD analysis, and the chemical fingerprints were revealed at the wavelength 254 nm. The identification of ferulic acid (A), cAMP (B), calycosin (C), formononetin (D), and 6-gingerol (E) were labeled here. Representative chromatograms were shown,* n* = 3.

Chemical standardization is to ensure the repeatability of herbal extract in all subsequent biochemical analyses. The amounts of major components were calibrated by a calibration curve derived from HPLC, which was obtained from a series of dilutions of the chemical markers. The calibration curve of ferulic acid was y=21.134x+19.607; calycosin was y=10.189x-10.129; formononetin was y=13.602x+12.705; cAMP was y=11.218x+55.42; and 6-gingerol was y=17.311x+25.1328 (Supplementary Table 1). In quality control of herbal mixture, 1 g of dried DBT_1155_ powder was proposed to contain 572.32 *μ*g of calycosin, 205.66 *μ*g of formononetin, 150.02 *μ*g of ferulic acid, 102.35 *μ*g of cAMP, and 1296.8 *μ*g of 6-gingerol. One gram of ZRR dried extract was proposed to contain 34.63 *μ*g of cAMP and 1203.24 *μ*g of 6-gingerol. These chemical requirements set the minimal standards. In addition, HPLC fingerprint was developed for the standardized extracts at 254 nm wavelength (Figure 1). These chemical parameters were employed as quality control to ensure the repeatability of biochemical assays.

### 3.2. Browning WAT Functions

**Figure 2:**
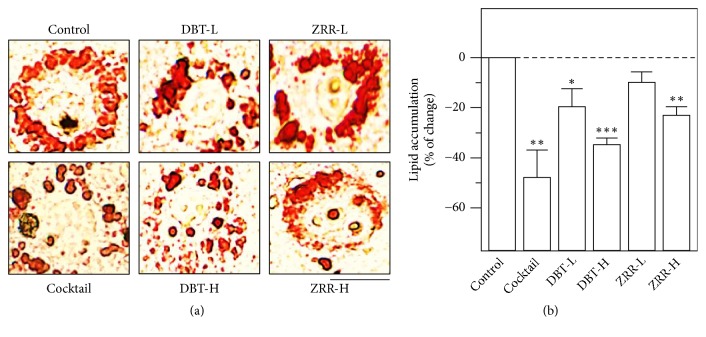
*DBT*_*1155*_ *decreases lipid accumulation*. 3T3-L1 adipocytes were cultured to 10 days of differentiation and then applied with cocktail (1.8 *μ*M of rosiglitazone and triiodothyronine), or different concentrations of DBT_1155_ (1 mg/mL of DBT labeled as DBT-H; 0.125 mg/mL of DBT labeled as DBT-L) or ZRR (1 mg/mL of ZRR labeled as ZRR-H; 0.125 mg/mL of ZRR labeled as ZRR-L) for another 3 days. (a) Oil Red O staining was to measure lipid accumulation. Bar = 50 *μ*m. (b) The amount of stained lipid was quantified at 490 nm absorbance. Data were expressed as mean ± SEM of the percentage of change as compared with control, where* n* = 3;* p *< 0.05 (*∗*);* p *< 0.01 (*∗∗*);* p *< 0.001 (*∗∗∗*).

The functions of DBT_1155_ and ZRR on lipid accumulation of cultured 3T3-L1 adipocytes were detected by Oil Red O. The optimized working concentration of DBT was determined by MTT assay; the highest working concentration of DBT_1155_ should be 1 mg/mL, which was labeled as DBT-H. The lowest concentration should be 0.125 mg/mL which was named as DBT-L (Supplementary Figure 1). The lipid accumulation was significantly decreased under application of DBT_1155_ extract, which was in a dose-dependent manner (Figures 2(a) and 2(b)). One mg/mL of DBT decoction (DBT-H) possessed ~35% decrease by lipid staining as compared to the negative control (Figures 2(a) and 2(b)). The antilipid accumulation effect, triggered by DBT_1155_, was much stronger than that of ZRR alone (Figures 2(a) and 2(b)). The lipid staining results implied that other constituents within DBT_1155_ might potentiate antilipid accumulation activity of ZRR. The IC_50_ of DBT_1155_ was ~0.375 mg/mL. In the same assay, the herbal extracts of AR, ASR, and JF did not show significant antilipid effect (Supplementary Figure 2). Here, the cocktail served as a positive control suppressing lipid accumulation dramatically by ~50% decrease, as compared with a negative control (Figures 2(a) and 2(b)).

**Figure 3:**
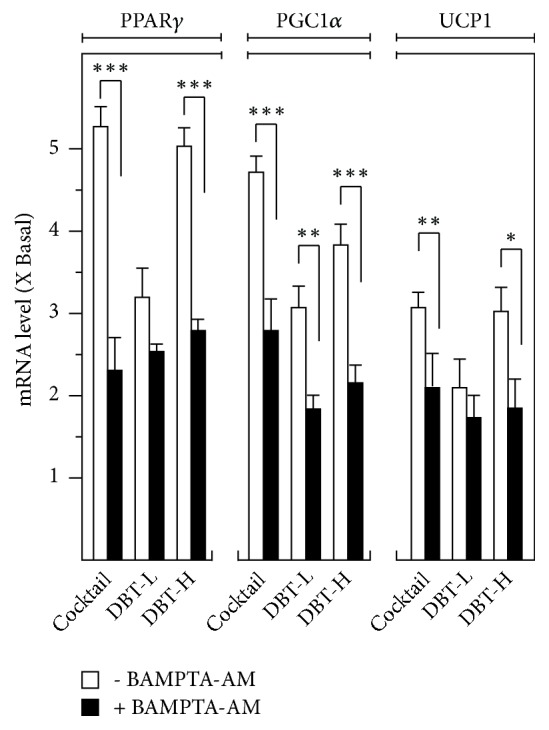
*DBT*_*1155*_ *triggers browning mRNA expressions of WAT markers*. 3T3-L1 adipocytes were cultured to 10 days of differentiation. Then, the cultures were applied with cocktail or different concentrations of DBT_1155_ (DBT-H: 1 mg/mL of DBT; DBT-L: 0.125 mg/mL) with/without cotreatment of BAMPTA-AM (10 *μ*M) for another 3 days. Total RNAs were isolated and reverse-transcribed to cDNA for PCR analysis. The mRNA levels of PPAR*γ*, PGC1*α*, and UCP1 were determined by the Ct-value method and normalized by the house keeping gene 18S rRNA. Data were expressed as mean ± SEM as compared with control, setting as 1 here, where* n* = 3;* p *< 0.05 (*∗*);* p *< 0.01 (*∗∗*);* p *< 0.001 (*∗∗∗*).

**Figure 4:**
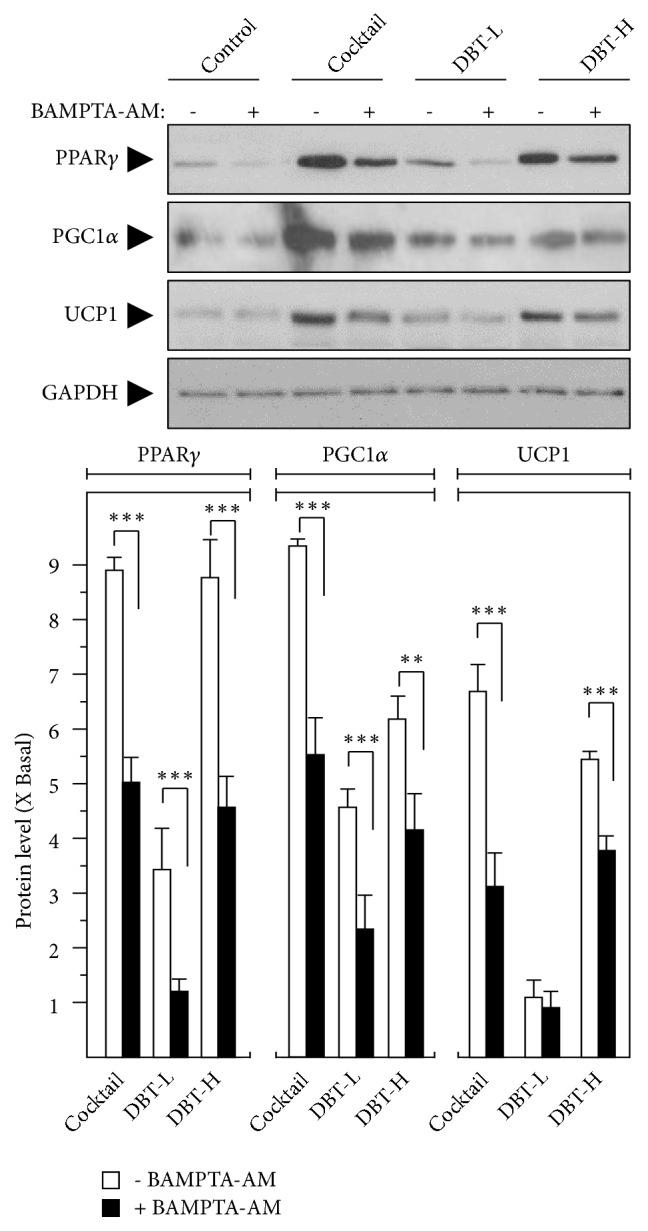
*DBT*_*1155*_ *triggers protein levels of browning WAT markers*. 3T3-L1 adipocytes were treated with cocktail or different concentrations of DBT_1155_ (DBT-H: 1 mg/mL of DBT; DBT-L: 0.125 mg/mL) with/without cotreatment of BAMPTA-AM (10 *μ*M) for another 3 days after 10 days of differentiation, as in Figure 2. The translational levels of PPAR*γ* (~58 kDa), PGC1*α* (~100 kDa), and UCP1 (~30 kDa) were detected by immunoblot analysis by specific antibodies. GAPDH (~38 kDa) served as an internal control. Quantification of target protein expression was calculated by a densitometer (lower panel). Data were expressed as mean ± SEM as compared with control, setting as 1 here, where* n* = 3;* p* < 0.05 (*∗*);* p *< 0.01 (*∗∗*);* p* < 0.001 (*∗∗∗*).

Increase levels of PPAR*γ*, UCP1, and PCG1*α* are the hall markers of WAT browning [25]. Indeed, the activations of these genes have been reported in obesity and/or its related diseases [25]. The transcript levels of these BAT-specific genes were revealed by RT-PCR from total RNA deriving from DBT_1155_-treated 3T3-L1 adipocytes. As shown in Figure 3, DBT_1155_ increased the mRNA levels of BAT markers in a dose-dependent manner. The maximal inductions of PPAR*γ*, PCG1*α*, and UCP1 were revealed at ~5-fold, ~4-fold, and ~3-fold, respectively, under the application of 1 mg/mL of DBT_1155_. Furthermore, calcium chelator, BAMPTA-AM, was employed here to identify the signaling pathway. The pretreatment of this chelator in 3T3-L1 adipocytes dramatically suppressed the BAT-specific gene transcription (Figure 3). The protein expression levels of these markers were also taken into consideration. The translational activities of these BAT-specific genes, e.g., PPAR*γ* at ~58 kDa, PCG1*α* at ~100 kDa, and UCP1 at ~30 kDa, were highly expressed, from 5-to-9-fold under the challenge of 1 mg/mL of DBT_1155_ (Figure 4). On the other hand, the application of BAMPTA-AM significantly abolished the increased protein expression, triggered by this ancient herbal formula (Figure 4). Taken together, DBT_1155_ decoction possessed antiobesity functions by accelerating WAT browning.

**Figure 5:**
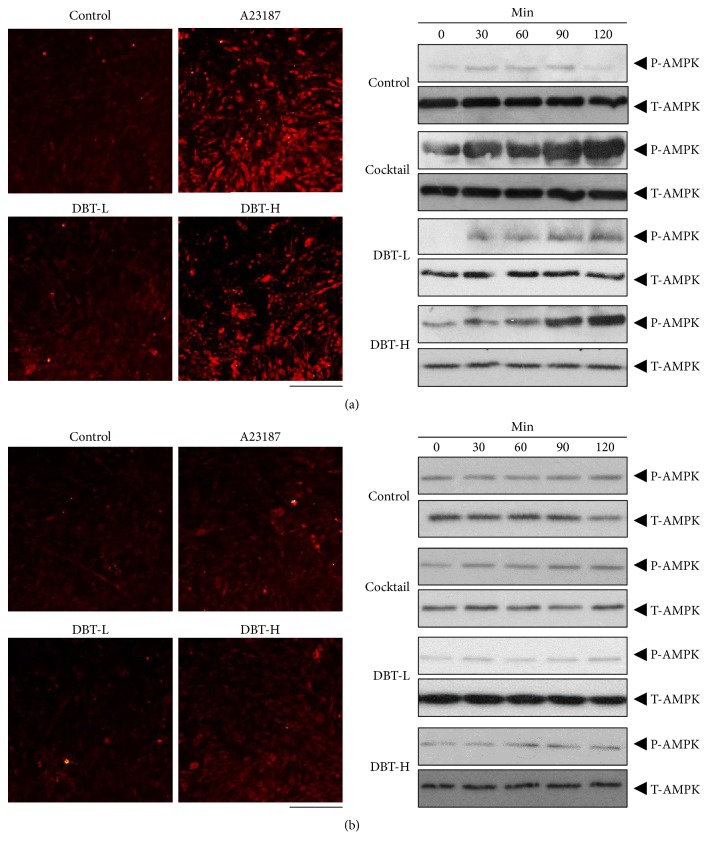
*DBT*_*1155*_ *stimulates Ca* ^*2+*^ *-AMPK pathway.* 3T3-L1 adipocytes were pretreated with medium (a) or BAMPTA-AM (10 *μ*M) (b) for 3 hours and then were labeled with fluorescent Ca^2+^ indicator Fluo-4 AM for half an hour. Fluorimetric measurement was performed after the treatment of different concentrations of DBT_1155_ decoctions, as in Figure 2. A23187 (100 nM) served as a control. The amounts of Ca^2+^ were evaluated by measuring the fluorescence intensity (left panel). Micrographs were taken by a confocal microscope; Bar = 100 *μ*m. Differentiated cells were subjected to the phosphorylation assay. Phospho-AMPK (P-AMPK, ~ 60 kDa) and total AMPK (T-AMPK, ~ 60 kDa) were revealed by using specific antibodies (right panel). Representative photos were shown,* n* = 4.

**Figure 6:**
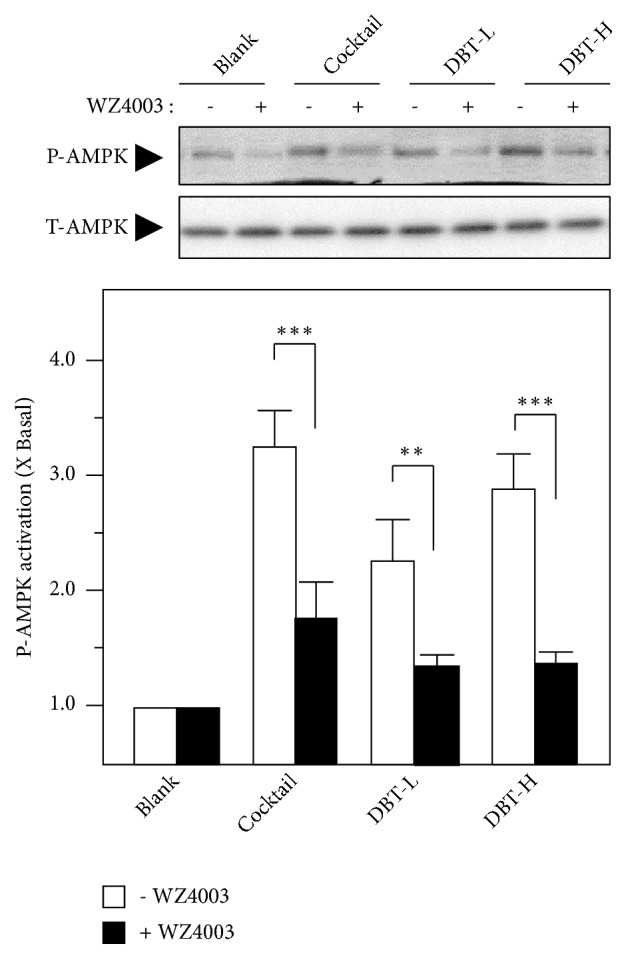
*WZ4003 suppresses AMPK phosphorylation*. 3T3-L1 adipocytes were pretreated with medium or WZ4003 (100 nM) for 3 hours and then subjected to the phosphorylation assay. The treatment of different concentrations of DBT_1155_ decoctions was as in Figure 2. Phospho-AMPK (P-AMPK, ~ 60 kDa) and total AMPK (T-AMPK, ~ 60 kDa) were revealed by using specific antibodies (upper panel). Quantification of protein expression was calculated by a densitometer (lower panel). Data were expressed as mean ± SEM as compared with control, setting as 1 here, where* n* = 3;* p *< 0.05 (*∗*);* p *< 0.01 (*∗∗*);* p *< 0.001 (*∗∗∗*).

AMPK signaling is a key player in regulating browning WAT. Application of DBT_1155_ in cultured 3T3-L1 adipocytes was capable of inducing AMPK phosphorylation, and this activation was in a time-dependent manner (Figure 5(a) right). The maximal stimulation was shown at 2 hours, as compared to control (Figure 5(a) right). Cellular Ca^2+^ level has been reported to be an indispensable factor regulating AMPK activities [26]. Here, the Ca^2+^ concentration in the treated 3T3-L1 adipocytes was detected by confocal microscopy. Fluo-4 AM, a Ca^2+^ indicator, was applied onto the cultures as to monitor the variation of Ca^2+^-induced fluorescence signal in differentiated 3T3-L1 cells. The increased Ca^2+^ level was found after the treatment in 3T3-L1 adipocytes (Figure 5(a) left). A23187, a calcium ionophore, served as a positive control (Figure 5(a)). In line with the above BAT-specific gene expression results, the pretreatment of BAMPTA-AM markedly suppressed the Ca^2+^ influx and AMPK activation in cultured 3T3-L1 cells (Figure 5(b)). Moreover, the pretreatment with WZ4003, a specific AMPK antagonist, reduced the phosphorylation of AMPK, as shown in Figure 6. Accordingly, these data indicated that DBT_1155_ triggered WAT browning in adipocyte via an AMPK signaling.

### 3.3. Fatty Acid Catabolism Activities

**Figure 7:**
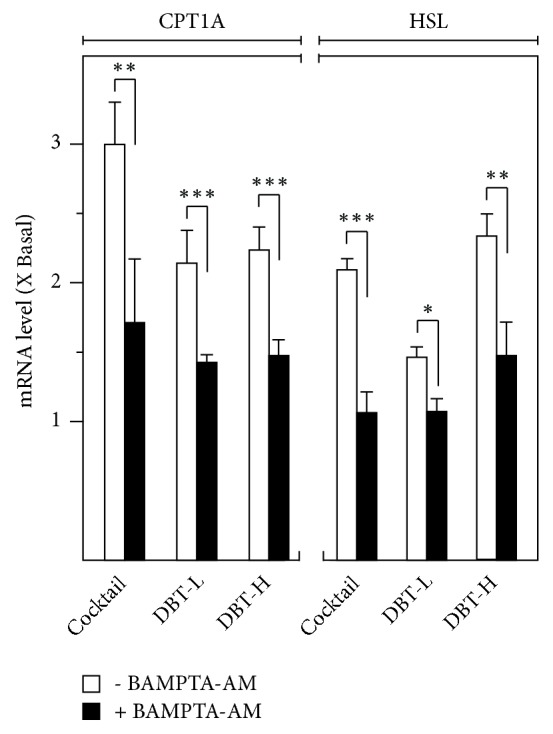
*DBT enhances fatty acid catabolism*. 3T3-L1 adipocytes were cultured for 10 days of differentiation. Then cocktail or different concentrations of DBT_1155_ with/without cotreatment of BAMPTA-AM (10 *μ*M) were applied for another 3 days, as in Figure 2. Total RNAs were isolated and reverse-transcribed to cDNA for PCR analysis. The transcriptional levels of CPT1A and HSL were determined by the Ct-value method and normalized by 18S rRNA. Data were expressed as mean ± SEM as compared with control, setting as 1 here, where* n* = 3;* p *< 0.05 (*∗*);* p *< 0.01 (*∗∗*);* p *< 0.001 (*∗∗∗*).

The key function of fatty acid catabolism is to generate ATP, and thus fat oxidation is a key switch to reveal catabolism progress [11, 27]. CPT1A is recognized in precipitating mitochondrial activities and accelerating fatty acid oxidation [26]. The transcriptional activates of these marker genes were revealed here (Figure 7). Treatment with high dosage of DBT_1155_ (1 mg/mL; DBT-H) led to significant increase of mitochondrial CPT1A mRNA, indicating the accelerated fat oxidation under the challenge of herbal decoction (Figure 7). Synthesis of fatty acid is another key regulator to modulate catabolism [26]. Overexpression of HSL was observed in the DBT_1155_-treated 3T3-L1 adipocytes. The maximal stimulation of HSL was revealed at ~2-fold, as compared with the control. The upregulation of HSL indicated that DBT_1155_ could have the possibility of suppressing fatty acid synthesis in 3T3-L1 adipocytes (Figure 7). Again, the mRNA levels of CPT1 and HSL in cultured 3T3-L1 adipocytes were downregulated upon pretreatment of BAMPTA-AM. Our data shed light on the antiobesity functions of DBT_1155_ via accelerating fatty acid oxidation and suppressing its synthesis.

## 4. Discussion

During the past decade, people paid attention to the study of weight loss therapy relying on herbal medicine. However, the side-effect of TCM in weight loss is unclear, which is one of the limitations in acceptance of TCM [21, 28]. This is an urgency to search for the safe TCM that could be effective for antiobesity. DBT_1155_ is a classical herbal decoction commonly used for treatment of “Blood” and “Qi” deficiencies. “Blood” in TCM theory is to provide nutrition. “Qi” is to boost antioxidative functions and finally neutralizes “Blood.” Indeed, this traditional herbal formula DBT_1155_ is comprised of 4 herbs, and each of them plays their specific responsibilities. AR is well-known for “Qi”-reinforcing, ASR is popular for “Blood-” nourishing, JF is famous for tonifying “Qi” and “Blood,” and ZRR is recognized to enhance vital energy as well as improving immune system in TCM theory [28]. DBT_1155_ was shown to upregulate erythropoietic genes* in vitro* and to reverse anemia-index in rats [21]. On the other hand, DBT_1155_ has been utilized for years, and the side effect has been rarely reported. Therefore, this herbal decoction should be safe to be consumed. Furthermore, the current data shed light on inducing brown fat phenotype in cultured 3T3-L1 via elevation of PPAR*γ*, UCP1, and PCG1*α* in both transcriptional and translational levels.

There are three types of bioactive constituents popular for obesity treatment. The first cluster is polysaccharide isolated from plant, acting as vital role in maintaining body health [28, 29]. In obesity animal models, the polysaccharides showed the possibilities of decreasing TG level via enhancing intestinal peristalsis, upregulating lipid absorption rate, and accelerating the transformation of exogenous cholesterol to bile acids [30]. For example, the total polysaccharide extracted from ginger had dual antiobesity functions by upregulating metabolic rate and inhibiting the absorption rate of calorie-dense dietary fats [31]. The JF-generated polysaccharide was capable of decreasing glycerol-3-phosphate dehydrogenase activity* in vitro* [32]. Flavonoid is the other bioactive group for obesity medication. Flavonoid increased thermogenesis via enhancing fatty acid transportation and reduced the triglyceride content in plasma and finally decreased lipid deposition [33]. In parallel, the* in vivo* working mechanism of flavonoids for losing weight has been well reported [34–36]. The intake of total flavonoids showed a possibility of alleviating obesity-triggered metabolic damage via suppressing inflammation [35]. Formononetin, one of isoflavones extracted from AR, as well as in DBT, was capable of stimulating AMPK pathway* in vitro*, and suppressed the development of obesity by attenuating high fat diet-induced body weight gain and visceral fat accumulation [36]. The last group of nature product for obesity treatment is believed to be polyphenols. Among them, the most famous one is curcumin [37–40]. Pan et al. demonstrated that treatment of male C57BL/6 J obese mice with curcumin significantly decreased body weight and fat mass after 2 months of observation, but enhanced insulin sensitivity in mice [37]. Moreover, oral administration of curcumin-enriched supplementation was effective in suppressing oxidative stress via modulation of antioxidation enzymes activities, i.e., superoxide dismutase (SOD) and glutathione peroxidase (GPx) in obese patients [39, 40]. The constituents of DBT_1155_ decoction, i.e., AR, ASR, and JF, showed synergistic effects to ZRR because the antilipid functions of AR, ASR, and JF were very limited. The cAMP-induced AMPK signaling is the major mechanism for antiobesity [41–44]. The DBT_1155_-triggered AMPK signaling could be significantly suppressed by the Ca^2+^ chelator; hence, the abovementioned data strongly supported that this conventional herbal formula reduced obesity by a Ca^2+^-AMPK signaling.

Obesity posts a great challenge on body health in a variety of ways, including high blood pressure and cholesterol, cardiovascular diseases, type II diabetes, and musculoskeletal discomfort [44]. The antiobesity functions of DBT_1155_ were never reported and, therefore, we believe this could be a significant breakthrough for further study. The aim here is to reveal TCM formulae that could be used for obesity treatment. Although our* in vitro* data suggest this herbal formula possesses antiobesity functions via accelerating WAT browning and lipid catabolism, the* in vivo* experiments are indispensable as to further confirm the functions.

## Data Availability

The data used to support the findings of this study are included within the article.

## Supplementary Materials

Supplementary Materials Supplementary Figure 1. Cell viability under the treatment of DBT1155. Supplementary Figure 2. ASR, AR, and JF show insignificant inhibition of lipid accumulation. Supplementary Table 1 Calibration curves, LOD and LOQ in HPLC analysis.
