# Supplementary figures and images for: An Ancient Chinese Herbal Decoction Containing Angelicae Sinensis Radix, Astragali Radix, Jujuba Fructus, and Zingiberis Rhizoma Recens Stimulates the Browning Conversion of White Adipocyte in Cultured 3T3-L1 Cells
Source: Evid Based Complement Alternat Med. 2019 Jun 16;2019:3648685. doi: 10.1155/2019/3648685 (PMC6601477; doi:10.1155/2019/3648685)

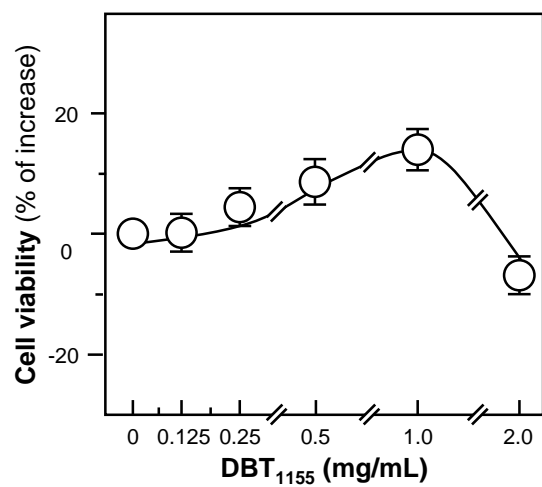

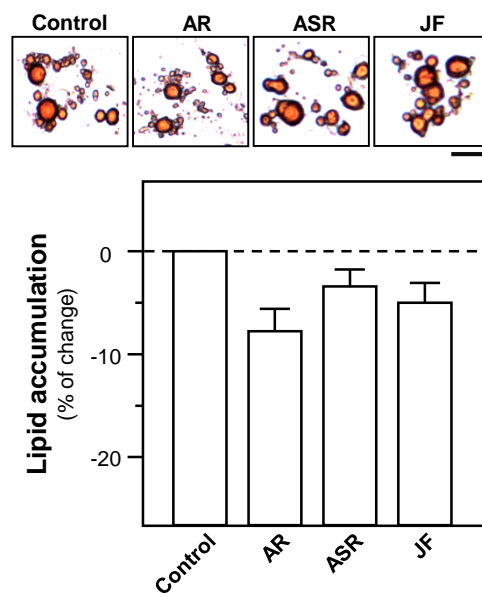

Supplement: Supplementary Materials — Supplementary Figure 1. Cell viability under the treatment of DBT1155. Supplementary Figure 2. ASR, AR, and JF show insignificant inhibition of lipid accumulation. Supplementary Table 1 Calibration curves, LOD and LOQ in HPLC analysis. [file 3648685.f1.zip › 3648685.f1/Supplementary Fig_ECAM_2785783.pdf]
