# Supplementary material for: An Ancient Chinese Herbal Decoction Containing Angelicae Sinensis Radix, Astragali Radix, Jujuba Fructus, and Zingiberis Rhizoma Recens Stimulates the Browning Conversion of White Adipocyte in Cultured 3T3-L1 Cells
Source: Evid Based Complement Alternat Med. 2019 Jun 16;2019:3648685. doi: 10.1155/2019/3648685 (PMC6601477; doi:10.1155/2019/3648685)
Supplement: Supplementary Materials — Supplementary Figure 1. Cell viability under the treatment of DBT1155. Supplementary Figure 2. ASR, AR, and JF show insignificant inhibition of lipid accumulation. Supplementary Table 1 Calibration curves, LOD and LOQ in HPLC analysis. [file 3648685.f1.zip › 3648685.f1/Sup Fig legend_ECAM_2785786.doc]

**Supplementary Figure 1. Cell viability under the treatment of DBT_1155_.**

3T3-L1 adipocytes were seeded in 96-well and cultured to 10 days of differentiation and then applied with various concentration of DBT_1155_ for another 3 days. MTT assay was to detect the cell viabilities. Data were expressed as mean ± SEM of the percentage of change as compared with control, where *n* = 4.

**Supplementary Figure 2. ASR, AR and JF show insignificant inhibition of lipid accumulation.**

3T3-L1 adipocytes were cultured to 10 days of differentiation and then applied with different herbal extract at 1 mg/mL (AR, ASR and JF) for another 3 days. Oil Red O staining was to measure lipid accumulation (upper panel). The amount of stained lipid was quantified at 490 nm absorbance (lower panel). Data were expressed as mean ± SEM of the percentage of change as compared with control, where *n* = 3.
