# Supplementary material for: An Ancient Chinese Herbal Decoction Containing Angelicae Sinensis Radix, Astragali Radix, Jujuba Fructus, and Zingiberis Rhizoma Recens Stimulates the Browning Conversion of White Adipocyte in Cultured 3T3-L1 Cells
Source: Evid Based Complement Alternat Med. 2019 Jun 16;2019:3648685. doi: 10.1155/2019/3648685 (PMC6601477; doi:10.1155/2019/3648685)
Supplement: Supplementary Materials — Supplementary Figure 1. Cell viability under the treatment of DBT1155. Supplementary Figure 2. ASR, AR, and JF show insignificant inhibition of lipid accumulation. Supplementary Table 1 Calibration curves, LOD and LOQ in HPLC analysis. [file 3648685.f1.zip › 3648685.f1/Supplementary Table 1_ECAM_2785787.doc]

**Supplementary Table 1 Calibration curves, LOD and LOQ in HPLC analysis.**

| Chemical | Calibration curve^a^ | Correlation coefficient (r^2^) | Linear range  (ng/mL) | LOD^b^  (ng/mL) | LOQ^c^  (ng/mL) |
| --- | --- | --- | --- | --- | --- |
| Ferulic acid | y=21.134x+19.607 | 0.9967 | 0.5-2000 | 0.26 | 0.85 |
| Calycosin | y=10.189x-10.129 | 0.9953 | 0.1-1000 | 1.24 | 3.73 |
| Formononetin | y=13.602x+12.705 | 0.9982 | 0.5-500 | 1.21 | 3.60 |
| cAMP | y=11.218x+55.42 | 0.9968 | 0.5-500 | 0.78 | 2.26 |
| 6-gingerol | y=17.311x+25.1328 | 0.9989 | 0.5-4000 | 0.37 | 1.07 |

^a^ The calibration curves were constructed by plotting the peak areas versus the concentration of each analyte. Each calibration curve included seven points.

^b^ LOD refers to the limits of detection.

^c^ LOQ refers to the limits of quantification.
